# The Waning of the Plague

**Published:** 1897-03-27

**Authors:** 


### THE WANING OF THE PLAGUE.

According to the published accounts there has been a considerable diminution in the number of cases of plague in Bombay, the reported deaths from that disease having fallen to 488 in the week, as against 843 a week only a month ago.

We would, however, again point out that the progress and virulence of the epidemic have all along been but imperfectly displayed by the official returns. Unless we are prepared to admit that, coincident with the plague, some other unrecognised epidemic has been going on, it is clear that the only way of obtaining anything like a true estimate of the mortality caused by plague is to compare the total death-rate during the epidemic with that of corresponding weeks in non-epidemic years; and when that is done we see that throughout the whole course of the epidemic there must have been a very considerable number of cases which have not only not been dealt with as plague during their lives, but have not even been recorded as plague when death has taken place.

According to people on the spot, who have watched the progress of events, we shall not be far wrong if we reckon the mortality from the plague as half as much again as that shown in the official returns. The practical importance of all this in forming an estimate of the comparative advantages of the various measures taken for the suppression of the disorder is manifest. A new *régime* is being introduced; the Government having taken the matter in hand, segregation of individuals is being enforced in addition to the cleansing of the city; and when the plague subsides, as it shortly will do, we shall doubtless hear much of the immediate impress made upon the epidemic by separating the sick from the healthy.

Now we have every sympathy with the principle of supplying hospitals for the treatment of infectious disorders; but it will not do to encourage illusions, and when we hear of plague being stopped by segregation, even if every reported case be isolated, we must remember the great number of cases which remain unreported and escape segregation altogether. Segregation is good so far as it goes, but we should be sorry to see credit claimed for it which really belongs to the efforts which have been made in the direction of the cleansing of the city, and to the diminution of the density of the population which has resulted from the general exodus of the inhabitants.

It is very difficult to judge to what extent this exodus has gone, but the Chamber of Commerce has estimated that in the months of November, December, and January, an excess of 358,852 persons left Bombay, and the *Times of India* considers that the total number of desertions cannot be less than 500,000, leaving a population of only about 300,000 in Bombay. If this is anything like a true estimate, it is clear that the virulence of the epidemic has been much greater than has generally been thought, the great loss of life being distributed among a much smaller number of people than the normal population of Bombay. Even calculated on the census population the death-rate of Bombay has risen to good deal more than 100 per 1,000 on several occasions this year, and if we are to believe that more than half the population had deserted, this rate must be more than doubled. If, then, we wish to understand what is meant by an epidemic of plague, even in this nineteenth century, in a civilised and progressive city, with town councillors and all the apparatus of modern municipal government in operation, we see that it means a loss of life equal to an annual death-rate of one in every ten of the census population, or one in every five of those estimated to have remained in the city!

